# Metabolic and pharmacological profiling of *Penicillium claviforme* by a combination of experimental and bioinformatic approaches

**DOI:** 10.1080/07853890.2022.2102205

**Published:** 2022-08-09

**Authors:** Zafar Ali Shah, Khalid Khan, Zafar Iqbal, Tariq Masood, Hassan A. Hemeg, Abdur Rauf

**Affiliations:** a Department of Chemistry, Islamia College Peshawar, Peshawar, Pakistan; b Department of Agricultural Chemistry & Biochemistry, The University of Agriculture, Peshawar, Pakistan; c Department of Medical Laboratory Technology, College of Applied Medical Sciences, Taibah University, Al-Medinah Al-Monawara, Saudi Arabia; d Department of Chemistry, University of Swabi, Anbar, Anbar, Pakistan

**Keywords:** *Penicillium claviforme*, metabolic profiling, molecular docking, pharmacological activities

## Abstract

**Background:**

*Penicillium* produces a wide range of structurally diverse metabolites with significant pharmacological impacts in medicine and agriculture. For the first time, a complete metabolome of *Penicillium claviforme* (*P. claviforme*) (FBP-DNA-1205) was studied alongside pharmacological research in this study.

**Methods:**

The metabolic profile of *P. claviforme* fermented on Potato Dextrose Broth (PDB) was investigated in this work. The complete metabolomics studies of fungus were performed using GC-MS and LC-MS-QTOF techniques. An *in vitro* model was utilised to study the cytotoxic and antioxidant activities, while an *in vivo* model was employed to investigate the antinociceptive and acute toxicity activities. Molecular Operating Environment (MOE) software was used for molecular docking analysis.

**Results:**

GC-MS study showed the presence of alkanes, fatty acids, esters, azo and alcoholic compounds. Maculosin, obtain, phalluside, quinoline, 4,4’-diaminostilbene, funaltrexamine, amobarbital, and fraxetin were among the secondary metabolites identified using the LC-MS-QTOF technique. The n-hexane fraction of *P. claviforme* displayed significant cytotoxic activity *in vitro*, with an LD50 value of 92.22 µgml^−1^. The antinociceptive effects *in vivo* were dose-dependent significantly (*p* < .001). Interestingly, during the 72 h of investigation, no acute toxicity was demonstrated. In addition, a docking study of tentatively identified metabolites against the inflammatory enzyme (COX-2) supported the antinociceptive effect in an *in silico* model.

**Conclusion:**

Metabolic profile of *P. claviforme* shows the presence of biologically relevant compounds in ethyl acetate extract. In addition, *P. claviforme* exhibits substantial antioxidant and cytotoxic activities in an *in vitro* model as well as antinociceptive activity in an *in vivo* model. The antinociceptive action is also supported by a molecular docking study. This research has opened up new possibilities in the disciplines of mycology, agriculture, and pharmaceutics.

**Key messages**
- The first time explored complete metabolome through GC-MS and LC-MS-QTOF.
- Both *in vivo* & *in vitro* pharmacological investigation of *P. claviforme*.
- *In silico* molecular docking of LC-MS-QTOF metabolites.

## 1. Introduction

The phylum Ascomycota includes the genus *Penicillium*, which is one of the most common and numerous groups of soil fungi. The genus *Penicillium* is classified into two subgenera and 26 sections within the Aspergillaceae, according to the International Commission on *Penicillium* and Aspergillus (ICPA), with 429 internationally recognised species. Mycologists and pharmaceutical industries have all been interested in structurally diverse secondary metabolites (SM) found in *Penicillium* species [1]. Some of the most important industrial and pharmaceutical SMs include Lovastatin (cholesterol-lowering drug) and penicillin (*β* lactam and the first broad-spectrum antibiotic). The production of a wide range of SMs has accelerated the process of drug discovery and development [2]. Recently, the drug development program has been accelerated to establish drugs by adopting a new approach called “repurposing” to treat new diseases. The toxicity and pharmacology of such molecules have already been established, allowing for the rapid translation of new indications into clinical practice [3].

Over the past two decades, the genus *Penicillium* has been extensively investigated for its biological, agronomic, biotechnological and pharmacological potential [4]. Polyketides [5,6], alkaloids [7,8], terpenoids [9,10] and lactones [11,12] are among the structurally diverse metabolites discovered in *Penicillium* sp. These compounds exhibited a variety of biological properties, including anti-HIV [5], cytotoxic [9,10], antioxidant [13] and antibacterial [7,13–15]. Patulin, a molecule isolated from *Penicillium*, has been established to be a natural lung cancer chemopreventive agent [16]. Similarly, antithrombotic, anti-inflammatory and antifungal drugs such as pravastatin and compactin have been documented from the genus *penicillium* [17]. The discovery of various commercially available drugs derived from microbial sources has elevated the importance of microbial research [18].

*P. claviforme* belongs to the section *Penicillium clavigerum*. It has well-developed and compact coremia and conidiophores, which distinguishes it from other species of the genus *Penicillium* [19]. Five recognised compounds were isolated from *P. Claviforme* in research, including cyclopeptin, m-hydroxybenzyl alcohol, isopatulin, 3-butyl-7-hydroxyphthalide, cyclopenine, and two new phthalides [20]. Similarly, three new andrastine derivatives from *P. vulpinum* have also been isolated and shown to have strong inhibitory activity [21].

The *Penicillium* genus is undoubtedly one of the largest and most well-known fungi among the various microbes. Keeping in view the broad pharmacological profile of the genus *Penicillium*, the aim of the current work was to investigate the complete metabolome of *P. claviforme* using LCMS-QTOF and GC-MS techniques. In addition, the pharmacological potential (*in vivo* and *in vitro*) of *P. claviforme* was extensively explored as well as an *in silico* molecular docking investigation, which will aid in the advancement of knowledge about this specie and its family, as well as pharmaceutical industries.

## 2. Methods and materials

### 2.1. Fungal strain and animals

The fungal strain *P. claviforme* (FCBP-DNA-1205) was obtained from the First Fungi Bank of the University of Punjab, Lahore, Pakistan [22]. Mice (BALB/c) 25–35 g, both male and female, were obtained from the National Institute of Health (NIH), Islamabad. Throughout the trial, they were kept on a 12 h/12 h light/dark cycle at 22 °C with free access to water and food. Appropriate NIH guidelines have been adopted for the care and use of these animals [23].

### 2.2. Fungal culture fermentation

A suspension of spores (10^5^ conidia/ml) of *P. claviforme* (FCBP-DNA-1205) was inoculated centrally into 20 litres of medium potato dextrose broth (PDB) (potato extract 4 gL^−1^, dextrose 20 gL^−1^) kept in 500 ml sterilised Erlenmeyer flask. The pH of the medium was adjusted to 5.6 ± 0.5. Inoculated flasks were maintained at 28 °C under static conditions for three weeks. A haemocytometer was used for spore counting [24].

### 2.3. Extraction and fractionation

A greenish mycelium with a pea-like odour was grown. The mycelium was dried, ground and extracted with ethyl acetate (EtOAc) (3 × 1.0 L). The solvent was evaporated, yielding a crude ethyl acetate extract (8 g), which was separated in a separating funnel with n-hexane (3 × 0.5 L). After removing the n-hexane layer and evaporating the solvent, an extract of 3 g was obtained. Both fractions were condensed under reduced pressure using a rotary evaporator (Buchi, Germany, Model R-300) [20].

### 2.4. Lcms QTOF analysis

Untargeted metabolic profiling of *P. claviforme* was performed with Agilent (1290 Infinity) coupled to a quadrupole time-of-flight (QTOF) and mass spectrometer (MS) (Agilent 6530). The positive ion mode was obtained by utilising the full scan mass spectrum. A Poroshell column (C18) was used for chromatographic separation (Agilent Technologies). The column was equilibrated for 40 min at a flow rate of 0.3 mlmin^−1^ in the mobile phase. The separation was performed using solvent A (0.1% formic acid + water + acetonitrile). In gradient elution mode, composition varied from 7–100% (0–15 min) to 100–7% (15 to 27 min). DAD was used as a detector to observe the absorbance from 210 to 635 nm. The QTOF-MS was set at a pressure of 35 psi. The capillary and fragmentor were set between 135 and 3500 V. The crude ethyl acetate extract was injected with the volume of 10 μL. The data obtained were provided access to the database of Personal Compound Database Library (PCDL) (Agilent Technologies, CA, USA) and METLIN based on high-resolution mass (MS/MS) [25].

### 2.5. GC-MS studies

The crude ethyl acetate extract of *P. claviforme* was run on an HP-5 (30 m × 250 m × 0.25 m) stationary phase column (Agilent 7890 A/5975C). EI mode was selected for the capillary column. The mobile phase, helium (carrier gas), was maintained at a flow rate of 1.2 mLmin^−1^. The retention indices helped in the identification of compounds, while the peak area provided information on the concentration of extrolites in the extract. The mass spectrum (EI) was investigated at 70 eV and between 35 and 650 amu. The mass spectrum obtained was compared to NIST 08.L mass spectrum library [26].

### 2.6. Brine shrimp lethality test

The ethyl acetate and n-hexane fractions of *P. claviforme* were analysed for cytotoxic activity. Brine solution was prepared at a concentration of 3 percent and Artemia’s eggs hatched in the brine under the continuous light source. Ten larvae were selected for each petri dish. The ethyl acetate fraction was added to Petri dishes at 100, 500 and 1000 gml^−1^, while the n-hexane fraction was added at 100, 500 and 1000 gml^−1^, respectively. Dimethyl sulfoxide (DMSO) was used as a negative control. After two days, the mortality rate of brine shrimp was observed in both the extracts and the control. The larvae which showed no movement after several seconds of observation were considered dead [27]. The LD50 µgml^−1^ of both extracts was calculated using non-linear regression in the GraphPad package 8.0.

### 2.7. DPPH radical scavenging assay

Percent radical scavenging activity was assessed using the DPPH protocol [28]. The scavenging activity of the ethyl acetate fraction at 25, 50, and 100 µgml^−1^, and the n-hexane fractions at 25, 50, and 100 µgml^−1^ were evaluated. Briefly, a 0.02% (w/v) solution of DPPH in methanol was prepared. 1.5 ml of the 0.02% methanolic DPPH solution were added to 1 ml each of different concentrations of both fungal fractions (n-hexane & EtOAc). The solution was kept in the dark for half an hour; the absorbance maxima were noted at 517 nm using a spectrophotometer (6800 UV-VIS spectrophotometer). Methanol was included as a blank. The percentage (%) of DPPH radical scavenging was measured using the following formula:

% RSA=100−[(As/Ac) ×100]

where, As = sample absorbance

Ac = negative control absorbance

### 2.8. Antinociceptive study

The antinociceptive study of crude ethyl acetate extract of *P. claviforme* was evaluated using acetic acid-induced abdominal constriction test [29]. The extract of *P. claviforme* at varying doses ranging from 50 to 150 mgkg^−1^ was administered orally; the diclofenac sodium (positive control) was also administered orally by intraperitoneal (i.p.) route at a dose range of 50 mgkg^−1^. After one hour, the mice were given the intraperitoneal injection of 1% acetic acid at a volume to mass ratio of 10 mlkg^−1^. Abdominal constrictions were counted and calculated after ten minutes of continuous acetic acid injection for the next 20 min. Abdominal contractions were reported across all groups. The percent antinociception was calculated using the following equation:

Antinociception (%) = (1 – C1/C2) × 100

C1 = number of writhes in the treated groups

C2 = number of writhes in vehicle (5% DMSO and 1% Tween80)

One-way ANOVA followed by Dunnett’s *post hoc* test was performed using GraphPad prism package 8.0.

### 2.9. Acute oral toxicity

The acute oral toxicity of the *P. claviforme* extract was studied in mice. Randomised groups of six animals (25–30 g) were formed and these animals fasted for 12 h. The ethyl acetate extract, dissolved in a normal saline solution (10–20 mgkg^−1^), was administered using feeding canola. The control or vehicle-treated group received the same quantity of normal saline. All the animals were provided free excess water. Manifestations of acute toxicity were observed in two phases, 06 and 72 h. Daily visual observation on overall health, mortality, growth, gross behaviour, morbidity and physical activity if exits were carefully documented [30]. The animal study was approved by an ethical committee, Department of Pharmacy, University of Peshawar, KP, Pakistan.

### 2.10. Molecular docking assay

To support antinociceptive activity, the compounds tentatively identified in ethyl acetate extract using LC-QTOF MS techniques were used for docking study against the inflammation supporting enzyme cyclooxygenase-2 (COX-2) (PDB ID: 5JVZ) [31]. The crystal structure of COX-2 was retrieved from Protein Data Bank (http://www.rcsb.org/pdb). Water molecules were removed and missing hydrogen atoms added to COX-2 before docking analysis; furthermore, the correct hybridisation state was assigned to each atom in each residue, and the correction of the charges was also performed using a preparation program embedded in the Molecular Operating Environment (MOE) software. The MOE software site finder tool was used for locating active residues at an active site of COX-2. Finally, the selected mycochemicals were docked inside the active pocket of COX-2 proteins, employing a docking program from MOE software. Thirty conformations were generated for each compound using selected torsion angles for all rotatable bonds. The binding energy for each COX-2 ligand complex system was calculated using the London Dock scoring function implanted in the MOE software [32,33].

## 3. Results and discussion

### 3.1. LCMS-QTOF

The integrated investigation of ethyl acetate extract of *P. claviforme* guide the rapid identification of 31 metabolites along with 13 unidentified extrolites mass peaks in the positive mode. Only peaks with a confidence level of 90 to 99% were selected. The noise signals were eliminated by running the sample in triplicate. Fungi in the reproductive stage build distinct devoted structures on their growth media in response to variations in environmental and physiological conditions, resulting in a significant difference in their ribosome, lipid, and protein composition. This affects the production of secondary metabolites in fungi [34]. The metabolites identified in this study will help to identify and distinguish among *Penicillium* spp through chemotaxonomy. The top 19 metabolites with a confidence level of 90 to 99% along with their corresponding details are provided in Table 1.

**Table 1.** LCMS-QTOF (positive mode) data of *P. claviforme.*

S. No	Name	Formula	RT	[M + H] ^+^m/z	Mass
1	Quinoline	C_9_ H_7_ N	2.406	130.0653	129.058
2	N-(6-Oxo-6H-dibenzo[b,d]pyran-3-yl)acetamide	C_15_ H_11_ N O_3_	2.516	254.0811	253.0739
3	5,8-tetradecadienal	C_14_ H_24_ O	15.724	226.2169	208.1831
4	Cetrimonium	C_19_ H_42_ N	16.892	284.3316	284.3321
5	Kurilensoside F	C_33_ H_58_ O_11_	17.112	648.4319	630.3981
6	3β,15β,17α-Trihydroxy-pregnenone	C_21_ H_32_ O_4_	21.898	719.4498	348.2305
7	5S-HETE di-endoperoxide	C_20_ H_34_ O_8_	17.565	425.2154	402.2263
8	Obtusin	C_18_ H_16_ O_7_	3.039	345.0972	344.0902
9	4,4′-Diaminostilbene	C_14_ H_14_ N_2_	2.965	233.105	210.1158
10	Amobarbital	C_11_ H_18_ N_2_ O_3_	1.942	227.1391	226.1319
11	4-(Trimethylammonio)but-2-enoate	C_7_ H_14_ N O_2_	0.943	144.1018	144.1024
12	Fraxetin	C_10_ H_8_ O_5_	3.09	209.0445	208.0372
13	Maculosin	C_14_ H_16_ N_2_ O_3_	2.026	261.1235	260.1163
14	Onchidal	C_17_ H_24_ O_3_	15.551	277.1803	276.1731
15	β-Funaltrexamine	C_25_ H_30_ N_2_ O_6_	3.561	909.4308	454.213
16	His Ile Leu	C_18_ H_31_ N_5_ O_4_	16.877	780.5102	381.2392
17	3-Hydroxybenzyl alcohol	C_7_ H_8_ O_2_	2.225	125.0601	124.0529
18	Linoleic acid	C_18_ H_32_ O_2_	19.298	281.2474	280.2401
19	Phalluside-1	C_41_ H_75_ N O_9_	24.091	726.5343	725.5449

**Figure 1.**
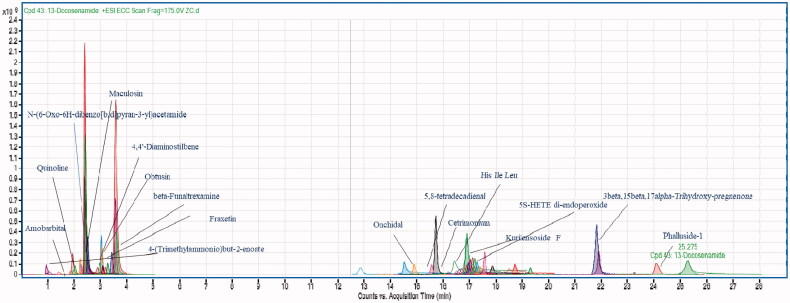
Metabolomic profiling using LC-MS QTOF (ESI^+^), the chromatogram of ethyl acetate extract of *P. claviforme* presenting molecular masses for different compounds.

The LC-MS chromatogram displayed peaks for all of the potential metabolites found in the mycelium of *P. claviforme* (Figure 1). Interestingly, the metabolites identified had previously been reported from different sources such as plants, microbes and marine organisms. Quinoline is a weak base with many pharmacological and biological activities [35]. These include analgesic, anthelmintic, cardiotonic, antifungal, anti-inflammatory, antimalarial, antibacterial and anticonvulsant activities. It is used as an intermediate in the synthesis of many products. Its higher exposure in mice has been linked to adverse effects [36]. N-(6-Oxo-6H-dibenzo[b,d]pyran-3-yl)acetamide belongs to coumarin class. A study was conducted to investigate the inhibitory action of acetamide coumarin on monoamine oxidase A in rats with serotonin using the radiometric protocol. The significant inhibitory response showed by coumarin metabolite [37]. Phalluside-1 is a glycolipid that has been isolated in large quantities from marine microorganisms. Significant antifungal activity has been shown by phallusides-1 against phytopathogenic fungi [38,39]. 3β,15β,17α-trihydroxy-pregnenone was synthesised by selective fungal specie through biotransformation. It showed promising activity against the inhibition of cholinesterase [40]. β-funaltrexamine is an alkaloid with morphinane nucleus. The inhibitory effect of morphine was blocked by β-funaltrexamine, demonstrating that it has a suppressive effect [41].

Anxiolytic and nicotine-induced effects were eliminated by the μ-opioid receptor antagonist -funaltrexamine (5 mgkg^−1^), suggesting a role in this behavioural response [42]. Obtusin is an anthraquinone derivative isolated from *cassia obtusefolia* that have significant antioxidant activity [43]. Obtusin was identified as larvicidal with LD50 of 1.7 ppm [44]. 4,4′-diaminostilbene is an azo compound with significant biological activities [45]. Amobarbital belongs to a class of barbiturates, and it is used as a hypnotic and sedative. In the epilepsy centre amobarbital is also advised to patients. Long-term use of this drug is prohibited due to dependency [46]. Fraxetin is the primary member of coumarin and is present in dietary supplements and functional foods. Its therapeutic properties have been reported as antiplatelet, antioxidant, hypoglycaemic, antibacterial and antiosteoporosis [47]. It was also recognised for inhibiting cystathionine synthase, with an IC50 value of 134 μM. Cystathionine β-synthase regulates human sulphur metabolism. Recent research has discovered that fraxetin plays a function in managing glucose levels in the kidney and liver, lowering the risk of type 2 diabetes [47]. 3-hydroxy benzyl alcohol is a phenolic compound that acts as a metabolite in various metabolic pathways. 3-hydroxy benzyl alcohol is reported for potent antileishmanial activity [48]. Maculosin is a phytotoxic compound that belongs to the diketopiperazine class, and it is used as a potent herbicide against knapweed [49]. Onchidal is a natural toxin that acts as an anti-acetylcholinesterase agent, and it regulates the transmittance of acetylcholinesterase enzyme at synapses [50]. It was previously reported using LCMS-QTOF analysis of dust containing different fungal species, including *Penicillium* [51].

Remarkably the m/z [M + H]^+^ 367.2388, 261.1103, 432.2263, 444.229, 736.4842, 692.4581, 604.4054, 560.38, 516.3534, 297.2399, 355.282 and 124.9643 did not show any match with available data of library database. The identification of pure compounds that correlate to these peaks will boost *P. claviforme's* value even more.

According to this research, *P. claviforme* (FCBP-DNA-1205) has a diverse metabolic profile. This study makes a significant contribution to the extrolites profiling of the *Penicillium* genus The results were interesting when compared directly to previously reported findings that several classes of metabolites had previously been reported from Penicillium strains, including polyketides, terpenes, amino acid derivatives, indole alkaloids, benzodiazepines, ergot alkaloids, quinoline alkaloids, and diketopiperazine [52]. A study performed on the *P. setosum* confirmed the presence of quinalizarin such as andrastin D in its metabolite pattern [34]. Patulin, a secondary metabolite found in several species of the genus *Penicillium*, was shown to be absent in *P. setosum* [53]. Patulin was not detected in our investigation, which added to the discrepancy. Although Patulin is thought to be a strong chemotaxonomic marker for identifying the genus *Penicillium*, phylogenetic investigations show that it is restricted to only a few *Penicillium spp* [54]. Our study agreed with the existing studies, based on the available data on the metabolomics of the genus Penicillium.

The crude extract and fractions of *P. claviforme* have shown significantly potent biological activities. The bioactive compounds produced by this pathogenic fungus were clearly shown by untargeted metabolomics research using tandem LC-MS/MS techniques such as quadruple/time-of-flight mass spectrometry (LC-MS-QTOF). This will help scientists in determining the bioactive as well as the toxic potential of *P. claviforme* in different domains to identify metabolites.

### 3.2. GC-MS analysis

**Figure 2.**
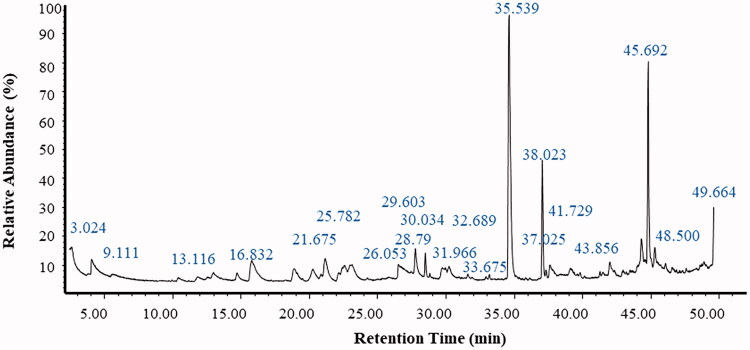
GCMS chromatogram of *P. claviforme.*

The volatile compounds in the ethyl acetate extract of *P. claviforme* were evaluated employing the GC-MS technique. This study was based on different retention times, peak areas and molecular mass (Table 2). A more important part of the fungal metabolome consists of volatile organic compounds (VOCs). In order to study the profiles of various volatile organic compounds of *P. claviforme* (FCBP-DNA-1205), the mycelium of fungus was subjected to GC-MS analysis. A well-defined total ion chromatogram (TIC) (Figure 2) revealed a transient profile of the strain. A total of 275 metabolites were identified with a retention time of about 1–50 min. These metabolites have been classified as fatty acids, organic acids, saccharides, nitrogenous compounds, simple long chain hydrocarbons and some typical GC impurities. *Penicillium* species are well known for the production of different types of fatty acids and carbohydrates [34]. The results indicated the presence of methyl esters of various fatty acids such as pentadecanoate, tridecanoate, 10-methyl decanoate, hexadecenoate, 9,12-octadecadienoate, 10,13-octadecadienoate and methyl 9-cis,11-trans-octadecadienoate. In addition, secondary metabolites of the alcoholic functional group such as behenic alcohol, n-pentadecanol, 1-henicosanol and cis-pinen-3-ol, as well as various carboxylic acids such as succinic acid, oxaloacetic acid, carbamic acid, benzoic acid, pentanedioic acid, benzeneacetic acid, nonanoic acid, decanoic acid and fumaric acid were obtained from the identified mycelium. These secondary metabolites are considered as an important tool for the characterisation and identification of fungi. However, very limited data on genus *penicillium* exist in this field. Similarly, different studies have been conducted on VOCs of *Penicillium* spp [34,55].

**Table 2.** Data obtained from GCMS spectrum of *P. claviforme.*

Retention time (minutes)	Area (Ab*s)	Match name	Molecular weight (amu)
26.053	19248287	1-Octadecene	252.282
28.79	10713339	Hexadecanoic acid, methyl ester	270.256
31.966	36914343	9,12-Octadecadienoic acid (Z,Z)-, methyl ester	294.256
21.675	9742818	1-Hexadecene	224.25
30.034	39221462	E-14-Hexadecenal	238.23
49.664	81708468	12-Oleanen-3-yl acetate, (3.alpha.)	468.397
16.832	1749715	1-Tetradecene	196.219
25.782	8359480	Benzyl Benzoate	212.084
		1-Nonadecene	266.297
29.603	9403164	1,2-Benzenedicarboxylic acid, butyl 2-methylpropyl ester	278.152
		Behenic alcohol	326.355
		1-Heneicosanol	312.339
33.675	40093240	1-Docosene	308.344
		3-Eicosene, (E)	280.313
37.025	2.5E + 08	Cyclotetracosane	336.376
		Propofol	178.136
		1-Hexadecanol	242.261
		1-Heptadecene	238.266
		1-Pentadecene	210.235
		n-Nonadecanol-1	284.308
		11-Tricosene	322.36
		n-Tetracosanol-1	354.386
		Cycloeicosane	280.313
32.689	1923942	Eicosane	282.329
		2(1H)Naphthalenone, 3,5,6,7,8,8a-hexahydro-4,8a-dimethyl-6-(1-methylethenyl)-	218.167

Although the existence of geosmin was mentioned in the literature, it was not detected in our study. However, the presence of its precursor farnesyl was confirmed in this study [56,57]. According to the published literature, the production of metabolites in fungi depends on various factors such as nutrients and temperature [58].

Our study evaluated the microbial VOCs of *P. claviforme*. Since microbial VOCs are known for their allergic, odour nuisance and genotoxic effects, their metabolic profiling will be extremely useful in medicine and the environment.

### 3.3. Cytotoxic activity

**Figure 3.**
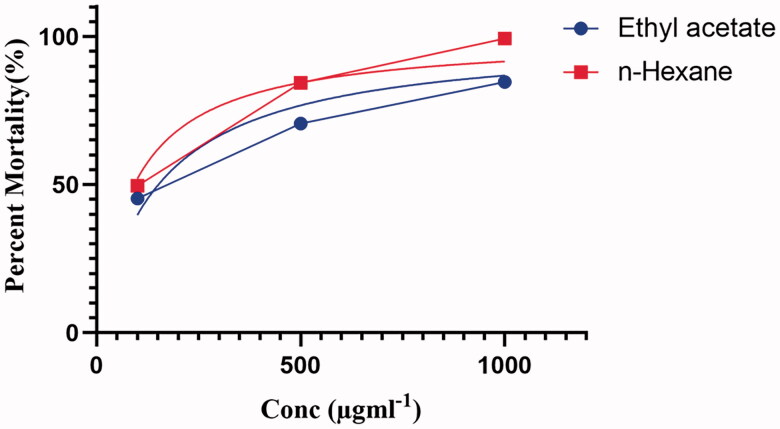
Percent mortality of Ethyl acetate and n-hexane extract of *P. claviforme.*

The genus *Penicillium* has the most robust and prolific cytotoxic potential among the fungi investigated, both qualitatively and quantitatively. *Penicillium* produces metabolites such as cyclopiazonic acid, gliotoxin, dicatenarin, secalonic acid D (SAD) and chatoglobosine, which influence cell division directly or indirectly and thus have a successful cytotoxic effect [59]. A secondary metabolite isolated from *Penicillium brefeldianum* (SD-273) was tested against Artemia salina, confirming an LD50 value of 9.4 μΜ [60]. Similarly, a metabolite of *Penicillium pinophilum* (SD-272), named 6,7-dihydroxy-3-methoxy-3-methylphthalide, showed a significant LD50 value of 11.2 M against *Artemia salina* [61]. The metabolites of *Penicillium* sp. (NTC-47) have high cytotoxic potential and were also assessed in an investigation [62]. In addition, the pigments isolated from *Penicillium simplicissimum* (DPUA 1379) and *Penicillium janczewskii* (DPUA 304) showed the highest mortality against *Artemia salina* [63]. Considering the cytotoxic effect of *Penicillium*, both ethyl acetate and n-hexane fractions of *P. claviforme* were tested for cytotoxicity against Artemia. The fungal extracts were evaluated at three different doses of (100, 500 and 1000) gml^−1^. Our study showed that the percent mortality was significantly higher at a dose level of 1000 gml^−1^ for both extracts (Figure 3).

The LD50 calculated for ethyl acetate and n-hexane extracts were 151.5 and 92.22 µgml^−1^, respectively (Table 3). The significant cytotoxic potency of the n-hexane fraction is supported by the presence of fatty acid molecules identified by GC-MS analysis. Previous studies have found that oils have high cytotoxicity against *Artemia*. This effect of the oil is attributed to them due to their role as free radical scavengers [64]. The preliminary study on the general toxicity of chemical substances has been carried out on Artemia for almost 30 years. This has helped the pharmacology industry in identifying target molecules for antitumor studies in the future [27].

**Table 3.** Percent mortality of extracts of ethyl acetate and n-hexane.

Dose (µgml^−1^)	% Mortality		
	Ethyl acetate	n-Hexane	Negative control
100	45 ± 0.42 c	50 ± 0.63 c	0
500	70 ± 0.33 b	85 ± 0.19 b	0
1000	85 ± 0.28 a	100 ± 0.77 a	0
LD50	151.5 µgml^−1^	92.22 µgml^−1^	–

All values are expressed as mean ± standard deviation (*n* = 3) *p* ≤ .05, LSD critical value for ethyl acetate 1.05 and 2.20 for n-hexane.

The findings of this investigation are consistent with previous research. The LCMS-QTOF and GC-MS analysis has revealed the presence of metabolites like fatty acids and alcoholic molecules, which could be a future candidate as cytotoxic and possibly be used in oncology.

### 3.4. DPPH radical scavenging activity

Oxidative damage is a major aetiological aspect implicated in numerous chronic human diseases such as cancer, diabetes mellitus, ageing, neurodegenerative diseases and atherosclerosis. Mushrooms are rich sources of antioxidant compounds that can help to reduce oxidative damage. It is reported in the literature that both the extract and the metabolites of fungi show potent radical scavenging effects, therefore, the antioxidant activity of both the ethyl acetate and n-hexane fractions was determined using the DPPH radical scavenging protocol [65]. A linear correlation was observed between the antioxidant potential of *P. claviforme* extract and the concentration of extract. The highest antioxidant potential was noted for 75 and 100 µgml^−1^ concentrations of ethyl acetate extract, with no significant variation in percent radical scavenging activity (%RSA). In the case of n-hexane fraction, the highest significant %RSA calculated was 65.03 at 100 µgml^−1^ (Table 4).

**Table 4.** Antioxidant activity of ethyl acetate and n-hexane extracts.

Sample	Concentration (µgml^−1^)	%RSA	
		Ethyl acetate	n-Hexane
*P. claviforme*	25	51.07 ± 0.02 c	44.94 ± 0.18 d
	50	62.56 ± 0.10 b	51.05 ± 0.20 c
	75	70.43 ± 0.04 a	58.98 ± 0.26 b
	100	75.03 ± 0.15 a	65.03 ± 0.09 a

All values are expressed as mean ± standard deviation (*n* = 3) *p* ≤ .05, LSD critical value for ethyl acetate 5.64 and 1.07 for n-hexane.

The display of the high antioxidant potential could attribute to the presence of metabolites such as kurilensoside F, cetrimonium, phalluside-1, obtusin, maculosin and onchidal. These metabolites can prevent oxidative damage by either scavenging oxygen, forming metal chelates, interfering with various oxidative processes, or detoxifying reactive oxygen species (ROS) by providing adequate oxidative defense and thus reducing the damaging effect on DNA, lipids and proteins [66].

Furthermore, it was found that the radical scavenging effect of ethyl acetate is stronger than that of n-hexane fractions; the high antioxidant activity of ethyl acetate extract is due to a high concentration or greater number of free radical scavenging molecules reported by LC-MS QTOF analysis.

### 3.5. Antinociceptive activity

Inflammation can cause discomfort and unpleasant sensations; research is underway to design, develop and formulate medications that would provide immediate relief to the infected or injured tissues. This study used crude ethyl acetate extracts of *P. claviforme* against the inflammation induced by the acetic acid in order to provide a quick reliever and evaluate the antinociceptive potential of *P. claviforme*. In addition, literature also reveals that endophytic and macrofungal extracts significantly decrease acetic acid-induced writhing in mice [67]. In an investigation, it was found that the methanolic mycelial extract of endophytic fungi significantly reduces writhing in mice induced by acetic acid [68].

**Figure 4.**
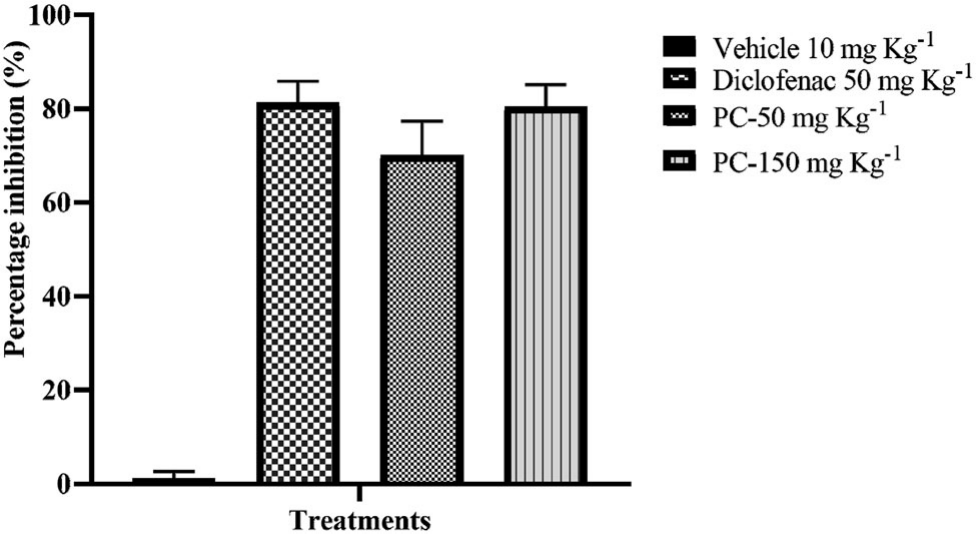
Percent Protection against ethyl acetate extract of *P. claviforme* at different concentrations.

Peritoneal induction of acetic acid stimulates the inflammatory response in mice. The prostaglandin biopathway is initiated and stimulates the sensation of pain, which becomes evident through a specific behaviour called writhing [69]. The acetic acid-induced writhing test demonstrating the antinociceptive activity of the ethyl acetate extract is shown in Figure 4. As expected, the control group showed the highest number of writhing. Administered doses of crude ethyl acetate extracts of *P. claviforme* (50–150 mgkg^−1^) strongly reduced acetic acid-induced nociception (*p* < .001) in a dose-dependent manner. This effect was clearly visible when calculating the percentage of antinociception. The most significant findings of this study were that the difference between the dose concentration of 150 mgkg^−1^ of ethyl acetate extract and 50 mgkg^−1^ diclofenac sodium (standard) is non-significant (Table 5).

**Table 5.** Antinociceptive effect of *P. claviforme* in the mouse abdominal constriction test.

Treatment	Dose (mgkg^−1^)	Number of writhes	Percentage inhibition (%)
Vehicle	10	32.83 ± 2.664	1.183 ± 1.520
Diclofenac	50	5.833 ± 1.276***	81.24 ± 4.637***
*P. claviforme*	50	9.833 ± 2.548***	70.15 ± 7.247***
*P. claviforme*	150	6.167 ± 1.249***	80.43 ± 4.701***

Data expressed as a mean number of writhes and percentage inhibition (%) ± SEM. ***P ˂ .001, as compared to the vehicle-treated animals group. One-way ANOVA followed by Dunnett’s *post hoc* test, *n* = 6 mice per group.

The present results suggest that *P. claviforme* extract could be a potent antinociceptive. The antinociceptive effect of *P. claviforme* extract could be attributed to the presence of different classes of compounds in ethyl acetate extract of *P. claviforme* such as anthraquinone, alkaloids, coumarin and glycolipids, all of which have been already reported for their antinociceptive activity [70].

### 3.6. Acute toxicity

The genus *Penicillium* has produced various mycotoxins. The lethality of fungal species depends strongly on mycotoxins [71]. Filamentous fungi produce a number of mycotoxins, including lethal ochratoxin A [72]. These mycotoxins induce their toxicity through the overexpression of HSP70, which causes apoptosis. It is believed that the availability or lack of various surrounding physiological factors strongly affects the production of metabolites [73]. The acute toxicity of two metabolites isolated from *Penicillium roqueforti* demonstrated toxicity in mice. Its mechanism is described as inhibition of ammonia and amino acid function [74]. A new mycotoxin was isolated from *Penicillium verruculosum*. These metabolites produced severe acute toxicity and tremors in mice, with an LD50 of 126.7 mgkg^−1^ [75]. Pigment isolated from *Penicillium resticulosum* was tested for acute toxicity in mice weighing between 500 and 1000 mgkg^−1^. These pigments were found to have low acute toxicity [76]. Considering the acute toxicity of metabolites as well as fungal extracts, the *P. claviforme* crude extract was subjected to an acute toxicity test on mice (Table 6).

**Table 6.** Acute toxicity of ethyl acetate extract of *P. claviforme.*

Treatment	Dose (mgkg^−1^)	Replication	Time interval (h)	Number of mice died
Negative	10	1	72	0/5
*P. claviforme*	10	1	72	0/5
	15	2	72	0/5
	20	3	72	0/5

The negative control showed no motility at a dose concentration of 10 mgkg^−1^. Same effect was observed for *P. claviforme* extracts in a 72-hour study at a dose ranging from 10 to 20 mgkg^−1^. The positive control showed significant mortality (100%), resulting in the death of all mice. Although *P. claviforme* metabolic profile identified several mycotoxins, their effects are not significant enough to result in mortality. The results suggest that different *Penicillium* species produce mycotoxins, but most of them are less toxic and are used in the fermentation of various foods [77].

### 3.7. *In silico* assay

**Figure 5.**
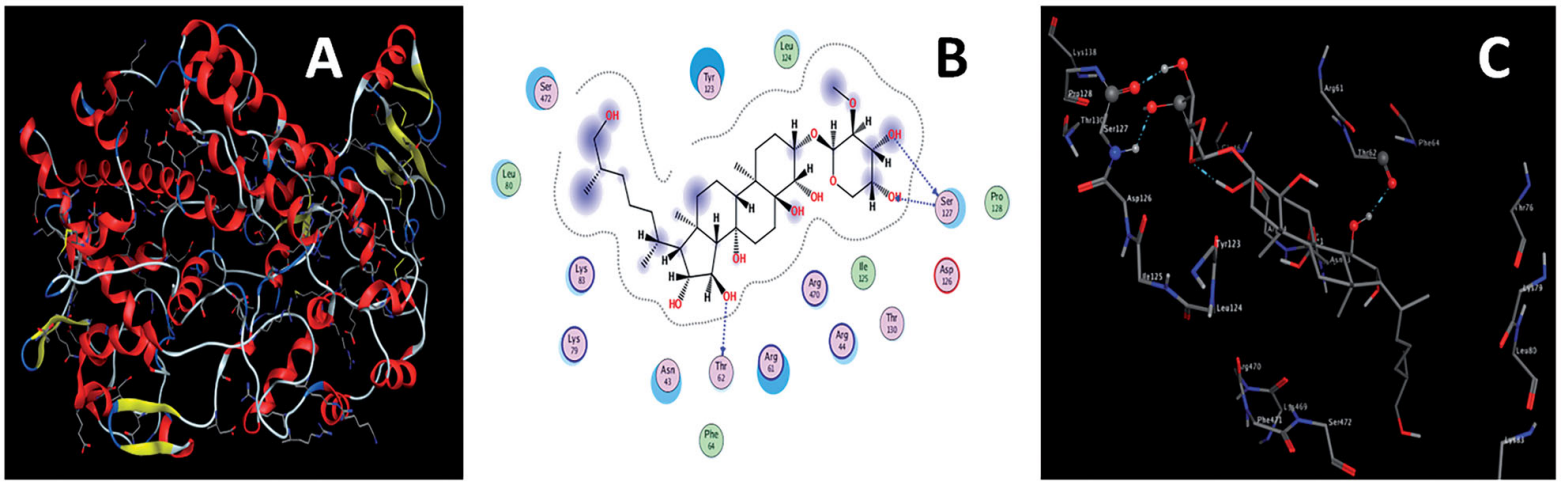
(A) 3 D structure of COX-2 (B) 2 D (C) 3 D interactions of L5 with COX-2.

Mycocompounds (L1-L12) tentatively identified in ethyl acetate extract were docked against COX-2 to support the antinociceptive activity of ethyl acetate extract of *P. claviforme*. Among the all ligands, L5 had the highest binding affinity of −9.4502 Kcalmol^−1^ (Figure 5) against COX-2.

L5 developed three H-bond interactions with residues Thr62, Ser127, and Ser127 of COX-2. One H-bond was generated between lone pair of electrons of the carbonyl oxygen atom of residue Thr62 and the polar hydrogen atom of the hydroxyl group of L5; a second H-bond originated between the polar hydrogen atom of the amino group of residues Ser127 and lone pair of electrons of the oxygen atom of the hydroxyl group of L5. The third H-bond formed between the polar hydrogen atom of the hydroxyl group of L5 and the lone pair of electrons of the carbonyl oxygen atom of residue Ser127. Interaction distances of 2.80, 2.79, and 2.96 A^0^ were documented for the first, second and third H-bond interactions, respectively (Table 7). Keeping in view the binding affinity, number of H-bonds and their interaction distances, L5 might form a stable L5-COX-2 complex system, and it may also be responsible for the inhibition of the activity of COX-2.

**Table 7.** Docking details of mycocompounds (L1-L14) against COX-2.

Ligands	Binding energy (Kcalmol^−1^)	Number of interactions	Nature of interactions	Interaction distance (A^0^)	Interacting residues
L1	−4.8130	02	pi-H	4.46	Tyr123
			pi-H	3.84	Phe372
L2	−5.7850	03	pi-H	4.29	Asp126
			pi-H	4.43	Asp126
			pi-H	3.84	Ser127
L3	−6.4789	01	H-acceptor	2.91	Arg61
L4	−7.0675	01	H-donor	3.29	Tyr123
L5	−9.4502	03	H-donor	2.80	Thr62
			H-donor	2.79	Ser127
			H-acceptor	2.96	Ser127
L6	−6.8173	03	H-donor	2.96	Asp126
			H-donor	2.81	Tyr123
			H-acceptor	3.14	Ser127
L7	−7.9987	03	H-donor	2.87	Ile125
			H-donor	2.72	Lys469
			H-acceptor	3.16	Ser127
L8	−6.6080	02	H-acceptor	2.98	Ser472
			H-acceptor	3.02	Arg44
L9	−5.8384	02	H-donor	3.03	Asn43
			H-donor	2.95	Lys469
L10	−5.1519	02	H-donor	2.98	Asn43
			H-donor	3.31	Lys469
L11	−4.4126	03	H-acceptor	3.00	Arg61
			H-acceptor	3.17	Arg61
			Ionic	3.39	Arg44
L12	−5.0901	02	H-donor	2.90	Tyr123
			H-acceptor	3.02	Ser127
L13	−6.0370	02	H-donor	3.21	Arg44
			H-acceptor	3.17	Ser127
L14	−6.4842	01	H-acceptor	3.08	Arg61

L1 = Quinoline, L2 = N-(6-Oxo-6H-dibenzo[b,d]pyran-3-yl)acetamide, L3 = 5,8-tetradecadienal, L4 = Cetrimonium, L5 = Kurilensoside F, L6 = 3β,15β,17α-trihydroxy-pregnenone, L7 = 5S-HETE di-endoperoxide, L8 = Obtusin, L9 = 4,4′-Diaminostilbene, L10 = Amobarbital, L11 = 4-(Trimethylammonio)but-2-enoate, L12 = Fraxetin, L13 = Maculosin, L14 = Onchidal

**Figure 6.**
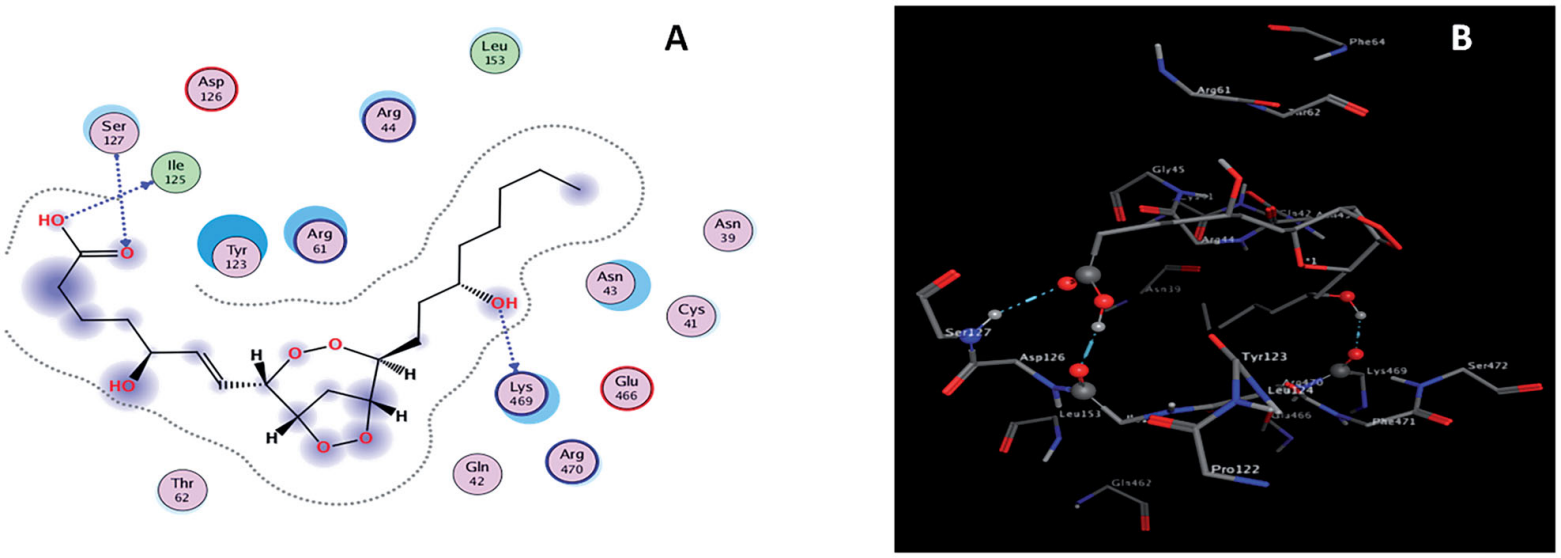
(A) 2 D (B) 3 D interactions of L7 with COX-2.

Similarly, L7 developed three H-bond interactions with COX-2 resulting in the second highest binding energy of −7.9987 Kcalmol^−1^. Physical interaction between the lone pair of electrons of the carbonyl oxygen atom of residue Ile125 and the polar hydrogen atom of the hydroxyl group of L5 was responsible for the formation of the first H-bond interaction (Figure 6); the lone pair of electrons of the oxygen atom of the hydroxyl group of amino acid Lys469 and polar hydrogen atom of the hydroxyl group of L7 were involved in the generation of second H-bond.

The third H-bond developed between the lone pair of the oxygen atom of the carbonyl group of L7 and the polar hydrogen atom of the amino group of residues Ser127. Interaction distances of 2.87, 2.72, and 3.16 A^0^ were recorded for the first, second and third H-bond interactions, respectively (Table 7). Docking data deduce that L5 might be used as a potential candidate for the inhibition of COX-2 due to its ability to the formation of stable L7-COX-2 complex system.

Similarly, docking results infer that the rest of the ligands also have a considerable amount of binding affinities, a reasonable number of H-bond interactions and effective interaction distances with the COX-2 enzyme resulting in the formation of the stable ligand-protein complex system. Considering the docking data these ligands might be potentially used against pathogenic protein COX-2. Docking details for the rest of the ligands are given in Table 7 and supplementary materials (Figure S2–S13). Results divulge that L5 and L7 are the most effective inhibitors among all ligands and that other mycocompounds can also form a stable ligand-protein complex system, corroborating the antinociceptive activity of ethyl extract of *P. claviforme*.

## 4. Conclusion

Fungi are rich sources of bioactive compounds with therapeutic and other important applications. For the first time, the metabolomic profile of *P. claviforme* was investigated using LCMS-QTOF and GCMS techniques. Biological relevant compounds such as maculosin, obtusin, phalluside, quinoline, 4,4′-diaminostilbene, funaltrexamine, amobarbital, fraxetin, alkanes, fatty acids, esters, azo and alcoholic compounds were tentatively identified. In a dose-dependent study, the n-hexane and ethyl acetate extracts were significantly potent as cytotoxic and antioxidant, respectively. Ethyl acetate extract of *P. claviforme* displayed strong antinociceptive action in an *in vivo* model supported by the presence of a non-significant difference in percent nociception between ethyl acetate extract and standard. Similarly, *P. claviforme* had no acute toxicity at any concentration, emphasising its relevance. *In silico* docking of LC-MS-QTOF metabolites with COX-2 enzymes supported the antinociceptive action of *P. claviforme*. The highest binding energy was calculated for the L5 ligand. However, more research is needed to isolate pure bioactive metabolites from *P. claviforme*, and determine their pharmacological potential and mode of action. As a result of this approach, fungi-based products for diverse applications will be developed.

## Supplementary Material

Supplemental Material

## Data Availability

The data that related to this paper are available on request from corresponding author.
