# Supplementary material for: Metabolic and pharmacological profiling of Penicillium claviforme by a combination of experimental and bioinformatic approaches
Source: Ann Med. 2022 Aug 9;54(1):2102–14. doi: 10.1080/07853890.2022.2102205 (PMC9367661; doi:10.1080/07853890.2022.2102205)
Supplement: Supplemental Material [file IANN_A_2102205_SM3622.doc]

**Supplementary Materials**

**Metabolic and pharmacological Profiling of *Penicillium claviforme* by a combination of experimental and bioinformatic approaches**

Zafar Ali Shah^1,2^, Khalid Khan^1^*, Zafar Iqbal^2^, Tariq Masood^2^, Hassan A. Hemeg^3^, Abdur Rauf ^4^*

1. Department of Chemistry, Islamia College Peshawar, Peshawar, KPK, Pakistan
2. Department of Agricultural Chemistry & Biochemistry, The University of Agriculture, Peshawar, Peshawar, KPK, Pakistan
3. Department of Pharmacognosy, Faculty of Pharmacy, Umm Al-Qura University, Makkah, P.O. Box 42, Kingdom of Saudi Arabia
4. Department of Chemistry, University of Swabi, Anbar, Anbar, KPK, Pakistan

**Table of contents**

1. **Figure S1**. Structure of docked ligands (L1-L14).
2. **Figure S2**. (A) 2D structure of COX-2 (B) 3D interactions of L1 with COX-2
3. **Figure S3**. (A) 2D structure of COX-2 (B) 3D interactions of L2 with COX-2
4. **Figure S4.** (A) 2D structure of COX-2 (B) 3D interactions of L3 with COX-2
5. **Figure S5.** (A) 2D structure of COX-2 (B) 3D interactions of L4 with COX-2
6. **Figure S6.** (A) 2D structure of COX-2 (B) 3D interactions of L6 with COX-2
7. **Figure S7.** (A) 2D structure of COX-2 (B) 3D interactions of L7 with COX-2
8. **Figure S8.** (A) 2D structure of COX-2 (B) 3D interactions of L8 with COX-2
9. **Figure S9.** (A) 2D structure of COX-2 (B) 3D interactions of L10 with COX-2
10. **Figure S10.** (A) 2D structure of COX-2 (B) 3D interactions of L11 with COX-2
11. **Figure S11.** (A) 2D structure of COX-2 (B) 3D interactions of L12 with COX-2
12. **Figure S12.** (A) 2D structure of COX-2 (B) 3D interactions of L13 with COX-2
13. **Figure S13.** (A) 2D structure of COX-2 (B) 3D interactions of L14 with COX-2
14. **Table S1.** Complete detail of LC-MS-QTOF analysis of *Penicillium claviforme*
15. **Table S2.** Complete detail of GC-MS analysis of *Penicillium claviforme*


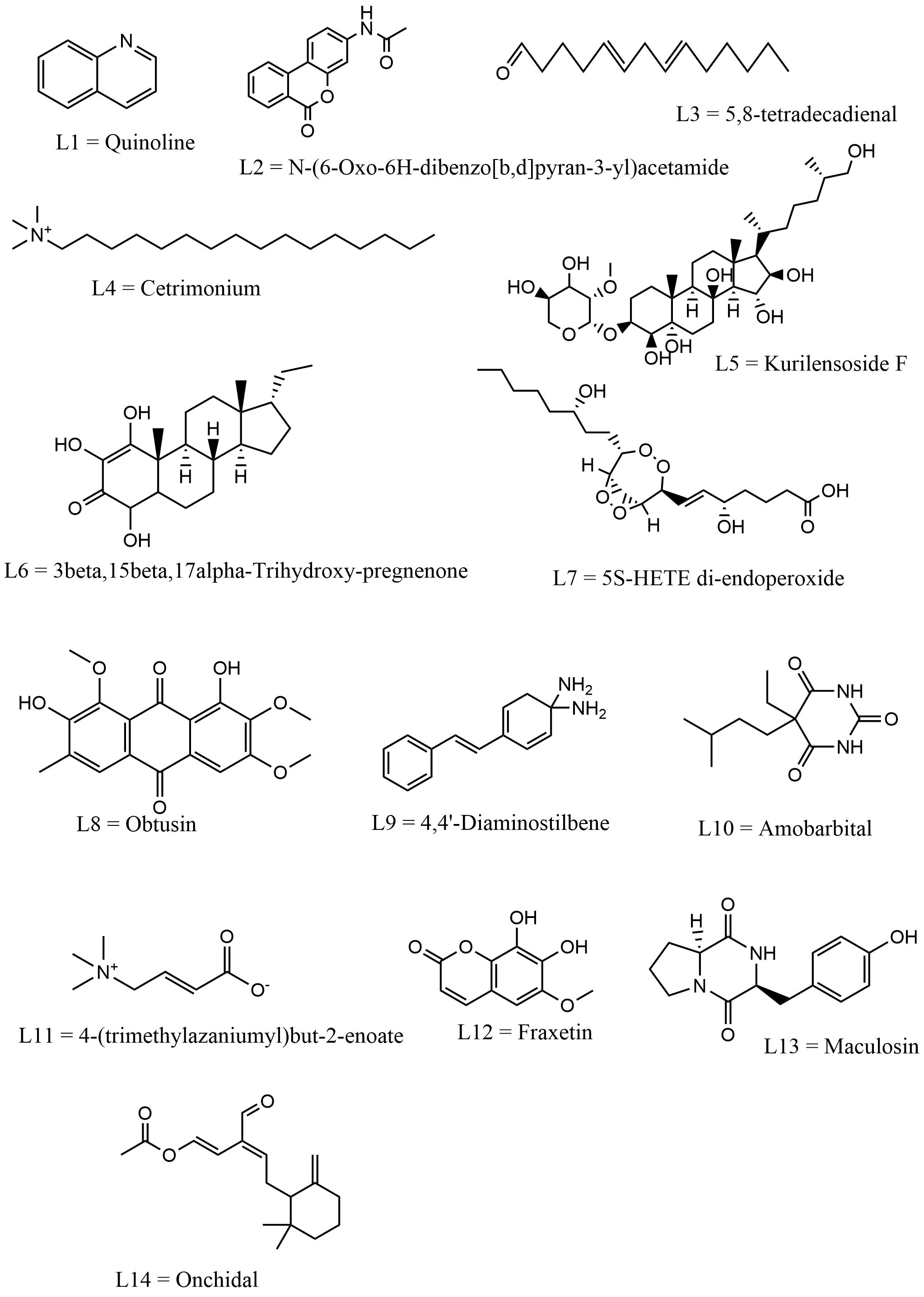


**Figure S1**. Structure of docked ligands (L1-L14).


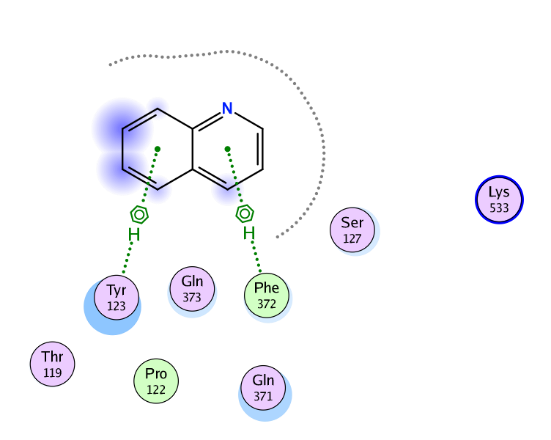

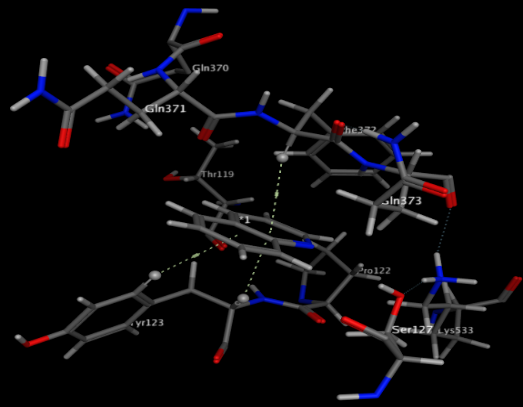


**A**

**B**

**Figure S2.** (A) 2D structure of COX-2 (B) 3D interactions of L1 with COX-2


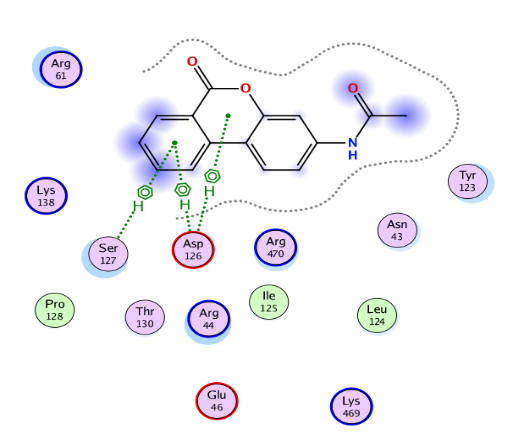

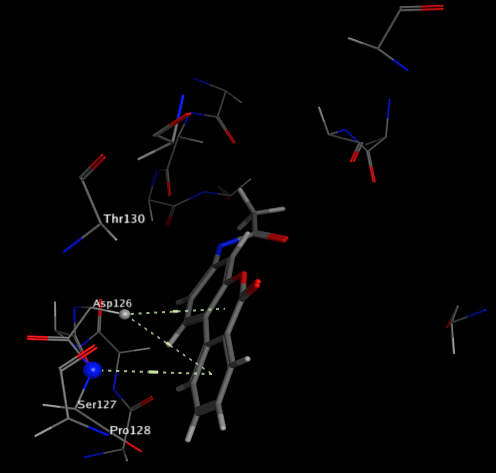


**A**

**B**

**Figure S3.** (A) 2D structure of COX-2 (B) 3D interactions of L2 with COX-2


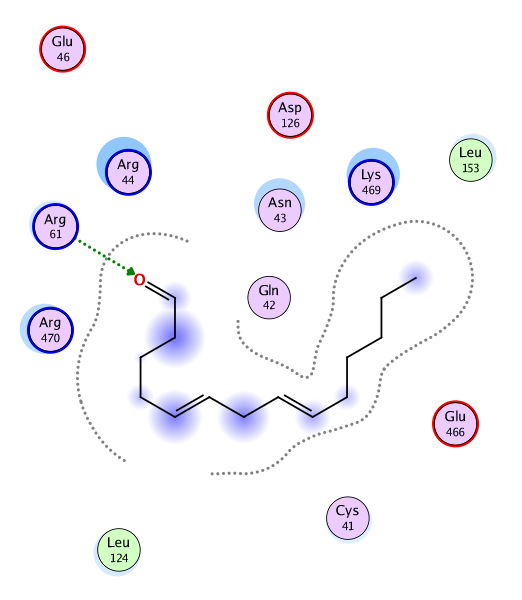

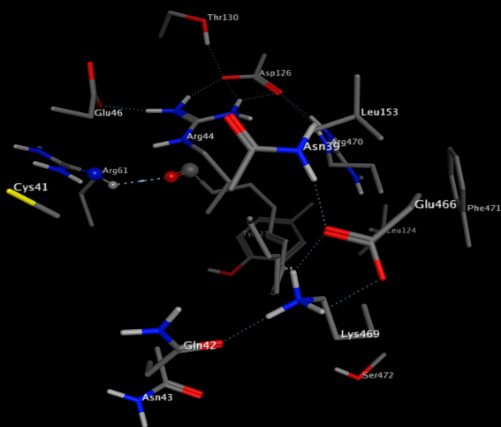


**A**

**B**

**Figure S4.** (A) 2D structure of COX-2 (B) 3D interactions of L3 with COX-2


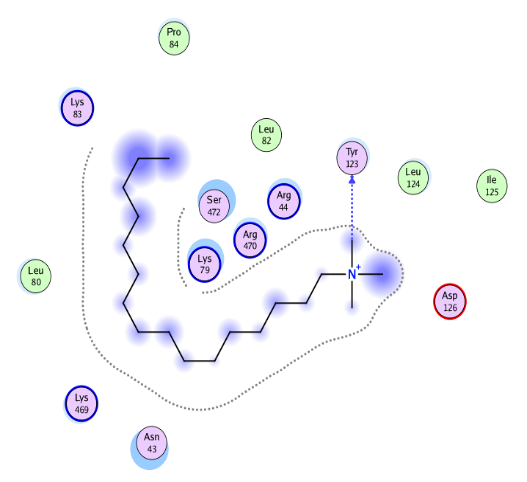

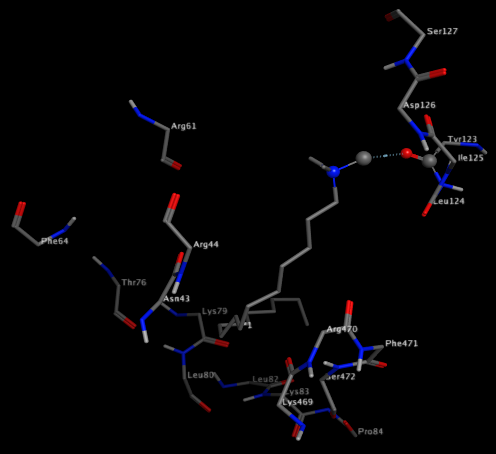


**A**

**B**

**Figure S5.** (A) 2D structure of COX-2 (B) 3D interactions of L4 with COX-2


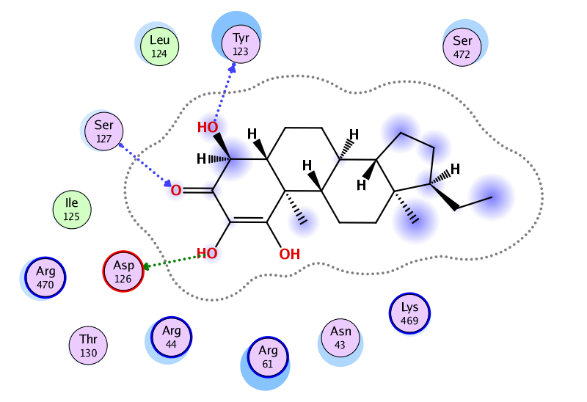

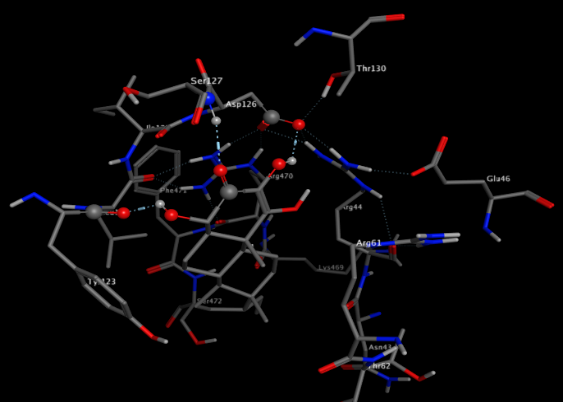


**A**

**B**

**Figure S6.** (A) 2D structure of COX-2 (B) 3D interactions of L6 with COX-2


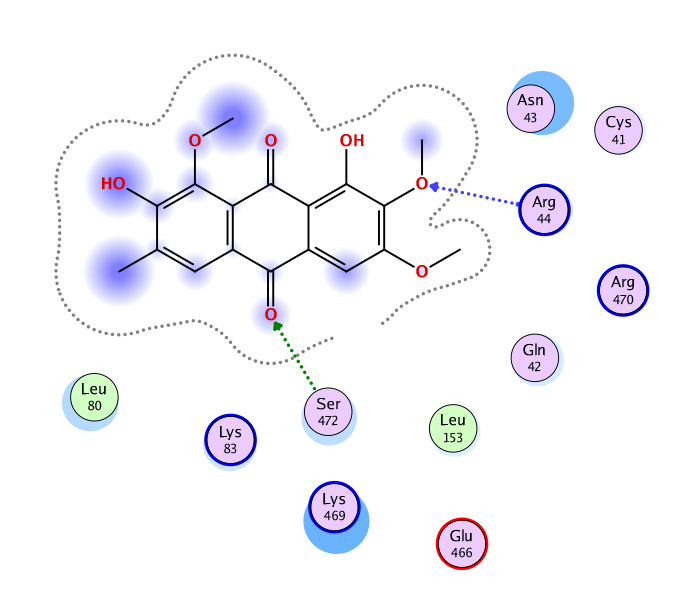

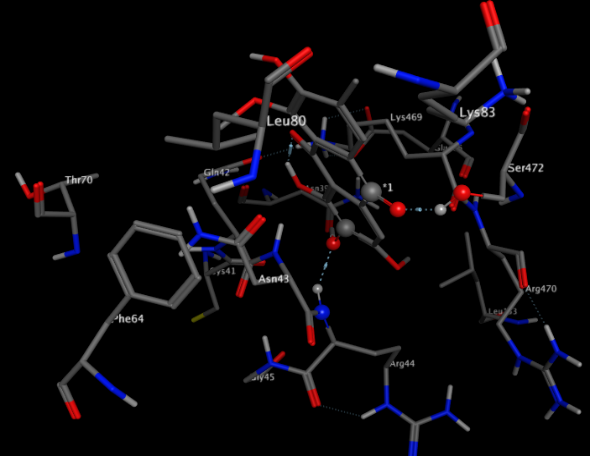


**A**

**B**

**Figure S7.** (A) 2D structure of COX-2 (B) 3D interactions of L8 with COX-2


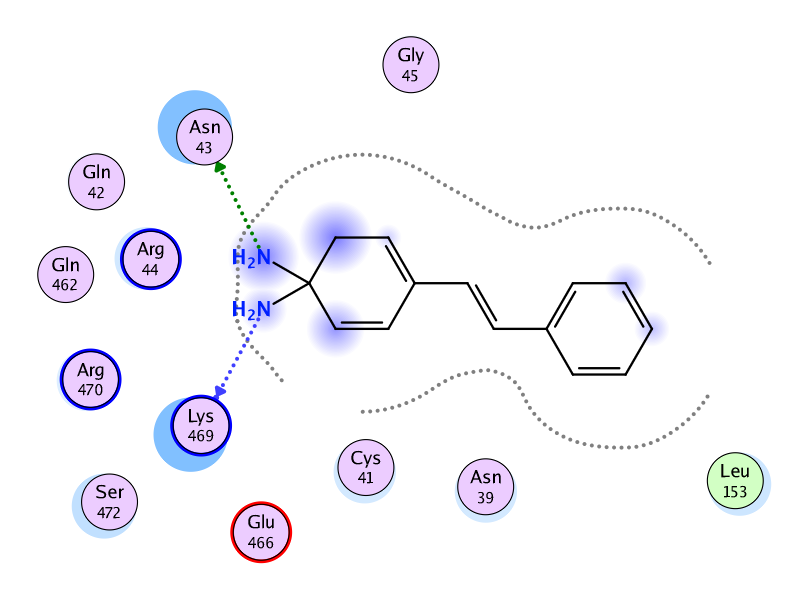

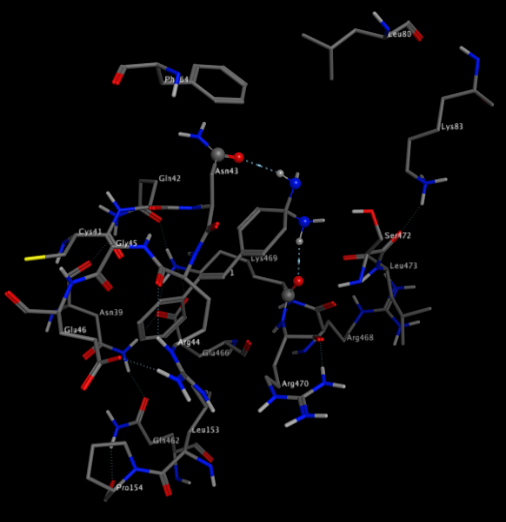


**A**

**B**

**Figure S8.** (A) 2D structure of COX-2 (B) 3D interactions of L9 with COX-2


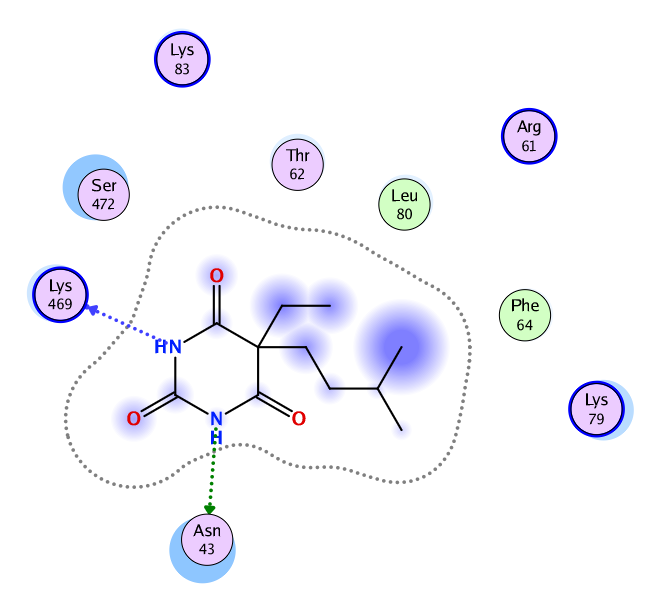

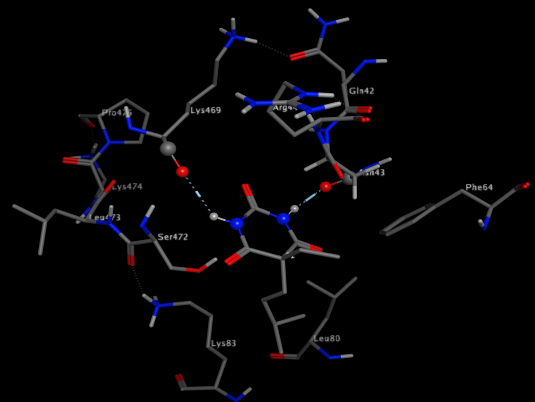


**A**

**B**

**Figure S9.** (A) 2D structure of COX-2 (B) 3D interactions of L10 with COX-2


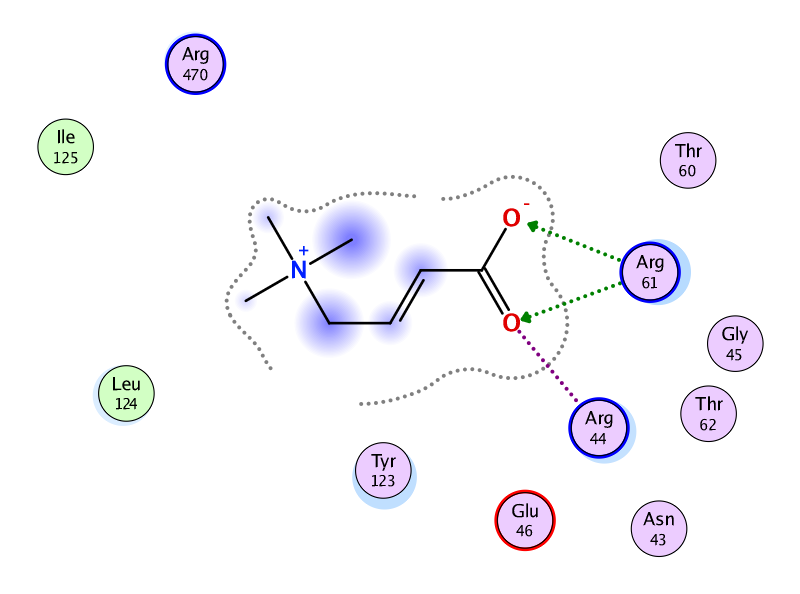

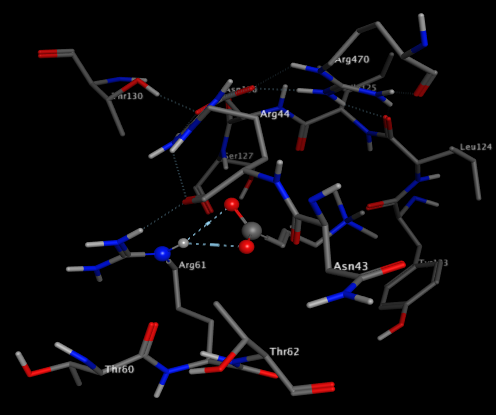


**A**

**B**

**Figure S10.** (A) 2D structure of COX-2 (B) 3D interactions of L11 with COX-2


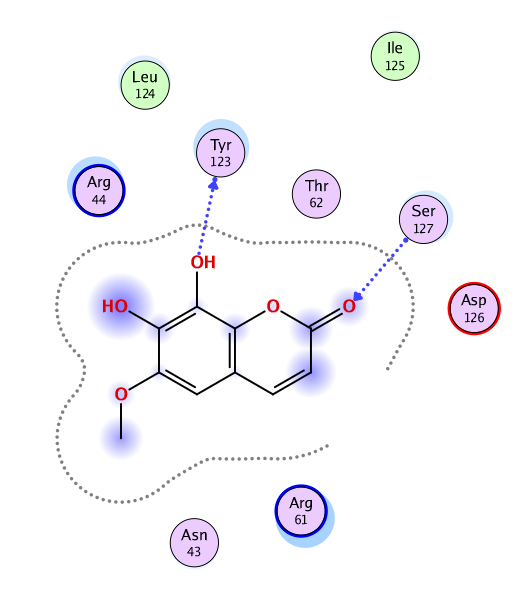

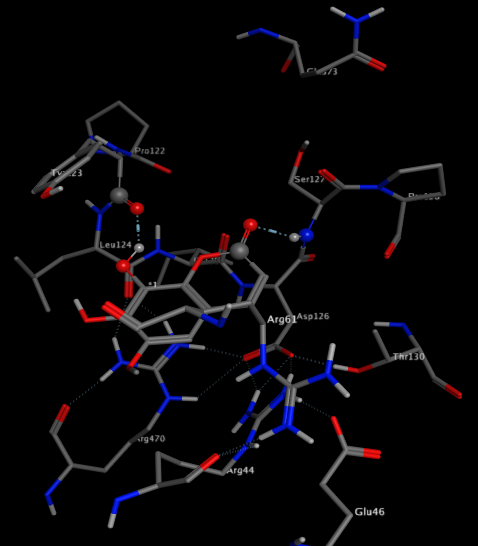


**A**

**B**

**Figure S11.** (A) 2D structure of COX-2 (B) 3D interactions of L12 with COX-2


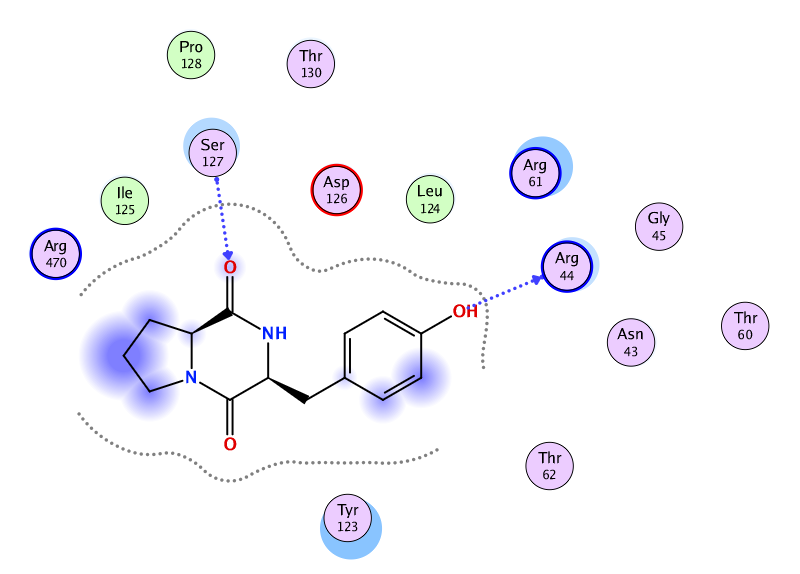

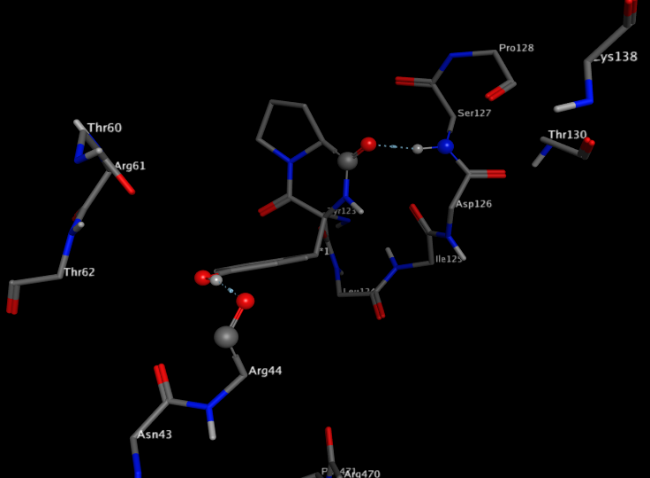


**A**

**B**

**Figure S12.** (A) 2D structure of COX-2 (B) 3D interactions of L13 with COX-2


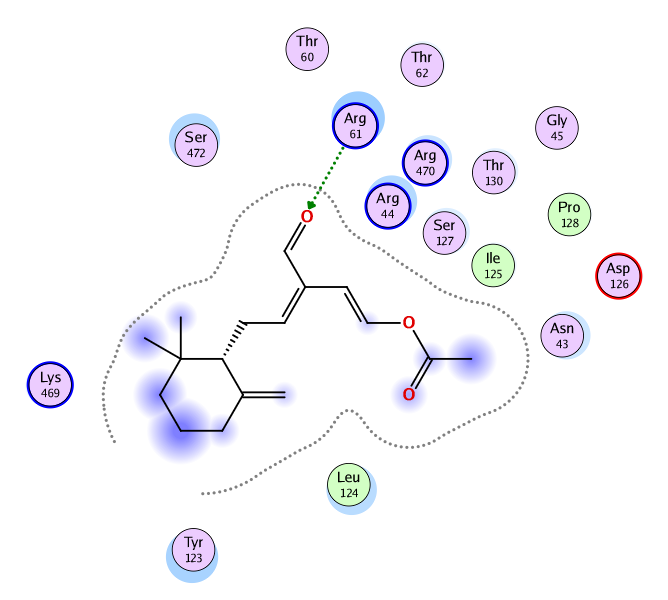

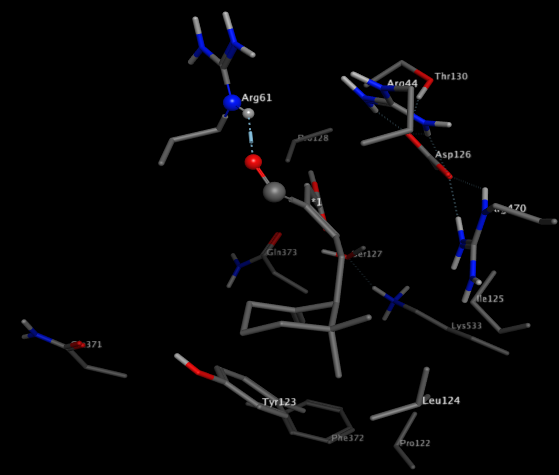


**A**

**B**

**Figure S13.** (A) 2D structure of COX-2 (B) 3D interactions of L14 with COX-2

**Table S1:** Original file provided in MS Excel as a separate supplementary material

**Table S2:** Original file provided in MS Excel as a separate supplementary material
